# Origin of domesticated water chestnuts (*Trapa bispinosa* Roxb.) and genetic variation in wild water chestnuts

**DOI:** 10.1002/ece3.10925

**Published:** 2024-02-07

**Authors:** Dinh Thi Lam, Taro Kataoka, Hiroki Yamagishi, Guoping Sun, Tetsuro Udatsu, Katsunori Tanaka, Ryuji Ishikawa

**Affiliations:** ^1^ Faculty of Agriculture and Life Science Hirosaki University Hirosaki Aomori Japan; ^2^ Faculty of Humanity and Social Science Hirosaki University Hirosaki Aomori Japan; ^3^ Zhejiang Provincial Research Institute of Cultural Relics and Archaeology Hangzhou China; ^4^ Faculty of Agriculture Miyazaki University Miyazaki Japan

**Keywords:** chloroplast genome, flow cytometry, genome content, maternal lineage, water chestnuts

## Abstract

The water chestnut *Trapa bispinosa* Roxb. has been domesticated in China and has been reported as the only domesticated species of this genus. To understand the origin of *T. bispinosa* and its evolution pathway, we compared the genetic similarity and seed morphology of domesticated water chestnut *T. bispinosa* with three wild species *T. natans*, *T. incisa*, and *T. japonica* along with archeological seed samples from the Tianluoshan site (approximately 7000–6300 cal BP) in China. The largest seed size was observed only in the domesticated species, whereas other wild species showed smaller size including *T. natans* L. genetically close to the domesticated type, and *T. incisa* was the smallest in size. The volumes of the seed capsule and endosperm were measured using X ray CT scans, showing the ratios of total volumes between *T. bispinosa* and wild species ranged from 4.2 to 4.5. The ratios of endosperm volume ranged from 3.3 to 3.7. Both measurements showed domesticated species have larger seed volume. Genome size was indirectly estimated by flow cytometry. Domesticated species with larger seed size was estimated as diploid, as were the wild species except for tetraploid species *T. japonica.* Domesticated species clearly showed the largest edible organs, but it was not a result of ploidy level changes. Maternal lineages traced using complete whole chloroplast sequences, suggested that *T. natans* is the closest to *T. bispinosa*, both of which are close to *T. japonica*. The result was confirmed by PCR genotyping with chloroplast insertion/deletion (cpINDEL) markers developed in the study. *T. incisa* showed distinct plastid types within the species, and *T. japonica* showed a unique plastid genotype. Our study concludes the largest volumes for the edible endosperm have been accomplished through nearly 6000 years of artificial selection, but the domestication did not involve ploidy level changes.

## Graphical abstract

Evolutionary history of ploidy level was confirmed among wild and domesticated water chestnut. Complete chloroplast genome sequences were applied to know maternal origin of domesticated species.

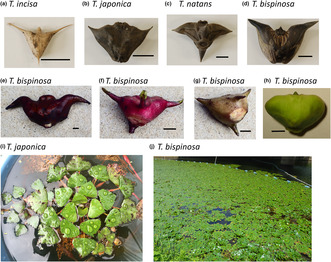

## 1 INTRODUCTION

Water chestnuts are a common name for aquatic plants belonging to Trapaceae family. Their distribution was preliminarily reported as the Old World (Cook, 1990) and widened through Asia, Africa, Australia, and America (Chen et al., 2007). The former water chestnut is predominantly cultivated in China (Guo et al., 2017) and is widely found in Eastern to Southeast Asian countries (Chiang et al., 2007; Puste et al., 2005; Singh et al., 2011; Suriyagoda et al., 2007; Takano & Kadono, 2005). The common species include *Trapa bispinosa*, *Trapa incisa*, *Trapa japonica*, *Trapa manshurica*, *Trapa natans*, *Trapa quadrispinosa*, and *Trapa taiwanensis*. Two species, *T. bispinosa* and *T. japonica*, are common species and easy to access for people in China and Japan. *T. bispinosa* is reported as the only domesticated species of this genus and is one of popular seasonal edible resources in China, Japan, Taiwan, and Vietnam, while *T. japonica* is a wild species that was collected by ancestral hunter‐gatherers as edible food at the beginning of the Jomon era found at the Kaihama‐toriduka site dated from about 12,000 BP and has been adopted as an edible wild plant in Japan to produce various processed foods (Sano et al., 2018). Although the productivity of *T. japonica* is lower than the domesticated species, it had been collected as edible resources during the Jomon prehistoric period (Arima et al., 1999; Suriyagoda et al., 2007). Nowadays, *T. japonica* in lakes and canals is generally regarded as a pest plant or as an indicator of the aquatic ecosystem (Fledman, 2001; Li et al., 2010; Sugimoto et al., 2014; Tsuchiya, 1983). *T. japonica* is cultivated in a restricted area of Japan because of the smaller size and low productivity compared to domesticated species (Hyakusima & Nakamura, 1974; Nakamura, 2004). Small‐sized type of water chestnuts were also collected during the Tianluoshan period in China (Guo et al., 2017). As the capsule of wild species is hard like walnut, the size may not change much over a long period. However, at present, larger larger‐sized water chestnut known as *T. bispinosa* is cultivated as a domesticated type from East Asia to South‐east Asia.

Speciation or phylogenetic relations of the *Trapa* genus are complicated and mostly depend on the morphological diversity such as the number of horns in the fruit. Some of them are synonymous like the case of *Trapa natans* which was defined as *T. quadrispinosa* (Li et al., 2017). Taxonomists previously utilized the number of spines on fruits. Generally, *T. bispinosa* and *T. quadrispinosa* /or *T. natans* have fruits with two and four barbed spines, respectively. However, local landraces of *T. bispinosa* show various morphological differences in the shape and number of spines (Nakano, 1964). Thus, these variations make biological relationships unclear due to lack of precise taxonomic standards for these morphological traits or molecular data. The size of fruits and the form of spines are not sufficiently precise criteria. For genetic studies, karyotype analysis had been adopted. Karyotype analysis indicated that chromosome numbers of both *T. natans* and *T. incisa* are 2n = 48 and that of *T. japonica* is 2n = 96 (Oginuma et al., 1996). In the genetic study, allozyme analysis suggested that hybridization between *T. natans* and *T. incisa* resulted in *T. japonica* (Takano & Kadono, 2005). Molecular markers are now in development to characterize taxonomic relations such as allozymes, rapid amplification of cDNA ends, chloroplast insertion/deletions (cpINDELs), nuclear single sequence repeats (SSRs), and amplified fragment length polymorphisms (Hoque et al., 2005; Jiang & Ding, 2004; Kim et al., 2010; Li et al., 2017; Takano & Kadono, 2005). Although molecular markers clearly distinguished *T. bispinosa* to other species, *T. quadrispinosa* which may be the same species as *T. natans*, and *T. japonica* from each other in the previous reports, the origin and evolutionary pathway still remain unclear. Recently, the whole chloroplast genome of *T. maximowiczii* has been reported (Xue et al., 2017). This sequence information will enable an increase in diagnostic markers to understand relationships.

In this study, we collected three wild species, *T. japonica*, *T. natans*, and *T. incisa* along with various landraces of the domesticated species, *T. bispinosa*: *T. japonica* present as a water chestnut used in the prehistoric Jomon period in Japan with a tetraploid genome. The species coexists with *T. natans* which is supposed to be *T. quadrispinosa*. *T. incisa* which only was found in Japan during our field trips. Those wild species along with domesticated species *T. bispinosa*. They inhabited commonly in Japan and China and rather than that no other species were observed during our field surveys. They were also selected to stand for ploidy level changes and suspected possible evolutionary relation of *Trapa* genus. Our study aims to: (1) understand the domestication pathway of water chestnuts; (2) develop molecular markers for tracing maternal lineages by the complete whole chloroplast genome sequence of *T. bispinosa*, and (3) give a clear picture of how wild species had been domesticated through human selection.

## 2 MATERIALS AND METHODS

### 2.1 Plant materials

In order to evaluate genetic variation, four species including *T. incisa* (*n* = 4), *T. japonica* (*n* = 17), *T. natans* (*n* = 10), and *T. bispinosa* (*n* = 53) were collected in China and Japan (Table S1). These materials collected comply with guidelines in both countries. Cultivated species were bought at local markets and wild species were sampled outside any restricted areas. Leaves and starch obtained from seeds were applied to extract DNA. Species were identified by the corresponding author, based on morphological traits. Only the species were coexisted in fields around the Tianluoshan site. Seed size was measured with multiple seeds from two populations for *T. incisa*, five populations for *T. natans*, 16 populations for *T. japonica*, and 22 populations for *T. bispinosa* (Table S2). In addition, two‐hundred and eighty‐six excavated seeds as archeological samples were applied for seed measurement, which were excavated at the Tianluoshan site (approximately 7000–6300 cal BP), Zhejiang province, China (Guo et al., 2017).

### 2.2 Next‐generation sequencing data

Edible water chestnuts, *T. bispinosa* and *T. japonica*, and two wild species *T. incisa* and *T. natans* were subjected to DNA extraction using a DNeasy Plant Mini kit (QIAGEN Co., Japan). Libraries had been prepared with a 350‐bp insert TrueSeq Nano DNA LT Sample Prep kit, 100‐bp pair‐end reads were obtained using a HiSeq 2500 system (Illumina Inc., Japan). The yields were 4605; 4657; 4552 and 4605 Mb, respectively. The number of reads in each case was 46,106,854; 45,598,236; 45,067,048 and 45,591,656, respectively. Percentages of more than Q30 quality (error rate < 10^−4^) were 94.62%, 94.59%, 95.20%, and 94.86%, respectively. De novo assembly was performed using the CLC Genomics Workbench (ver 8.0). Contigs with higher coverage and high similarity to the rice chloroplast genome were used to reconstruct chloroplast genomes. Re‐sequencing analysis was also attempted against the whole chloroplast genome of *T. maximowiczii* using the CLC Genomics Workbench (ver 8.0). Reads were screened for more than 200 coverages against the chloroplast genome, and more than 60% of presumed allelic INDELs in the chloroplast genome were extracted as a basic variant search. In addition to these INDELs, informative SNPs were compared to calculate similarity of genomes among three species.

### 2.3 INDEL markers genotyping

Nine INDEL markers were developed based on alignments among different genome sequences. PCR using these markers were amplified with a basic cycle of preheating at 94°C for 3 min, followed by 30 rounds of 95°C for 10 s, 55°C for 30 s, and 72°C for 30 s, and post‐heating at 72°C for 5 min with Thermopol *Taq* polymerase (NEB Ltd., Japan). The amplified DNA fragments were electrophoresed on 6% denaturing polyacrylamide gels at 1500 V for 2 h in 0.5 × TBE. The gels were then stained with silver nitrate.

### 2.4 X ray CT scanning

Alive samples of *T. insisa* (*n* = 3), *T. japonica* (*n* = 3), *T. natans* (*n* = 2), and *T. bispinosa* (*n* = 7), along with excavated samples (*n* = 3) which originated from T463‐7 block in the Tianluoshan site (Guo et al., 2017), were applied to X ray CT scanning. The volume of fruits was measured using a Bruker SkyScan 1172 scanner. Each sample was scanned at 50 kV, the scan depth was 800 μm and the resolution was 29.72 μm. After the capsules were scanned, the volumes of the capsule and inner endosperm were marked in contrasting colors. Three‐dimensional images were applied to distinguish the pericarp and inner spaces. The volumes of these two parts were extracted to estimate endosperm volume and volume of seeds from the image of inner space and image of the pericarp, respectively.

### 2.5 Phylogenetic tree

The number of base substitutions per site between sequences is shown in Table S3. Analyses were conducted using the Maximum Composite Likelihood model (Tamura et al. 2004). This analysis involved five nucleotide sequences. All ambiguous positions were removed for each sequence pair (pairwise deletion option), remaining a total of 156,067 positions in the final dataset. By using the informative nucleotides, evolutionary analyses were conducted in MEGA11 (Tamura et al., 2021). Parts of a rice single sequence copy from Nipponbare complete chloroplast genome X15901 were applied as an outgroup to construct the phylogenetic tree. Relatively high homology site from 105,059 to 113,727 nt in the single sequence copy was aligned with the corresponding sites of other Trapa species. Within the sequence, rice and *T. maximoviczii* shared 88.2% homology without gaps in which 5945 nt was not attempted and 2652 nt was matched with each other.

## 3 RESULTS

### 3.1 Seed size variation

**FIGURE 1:**
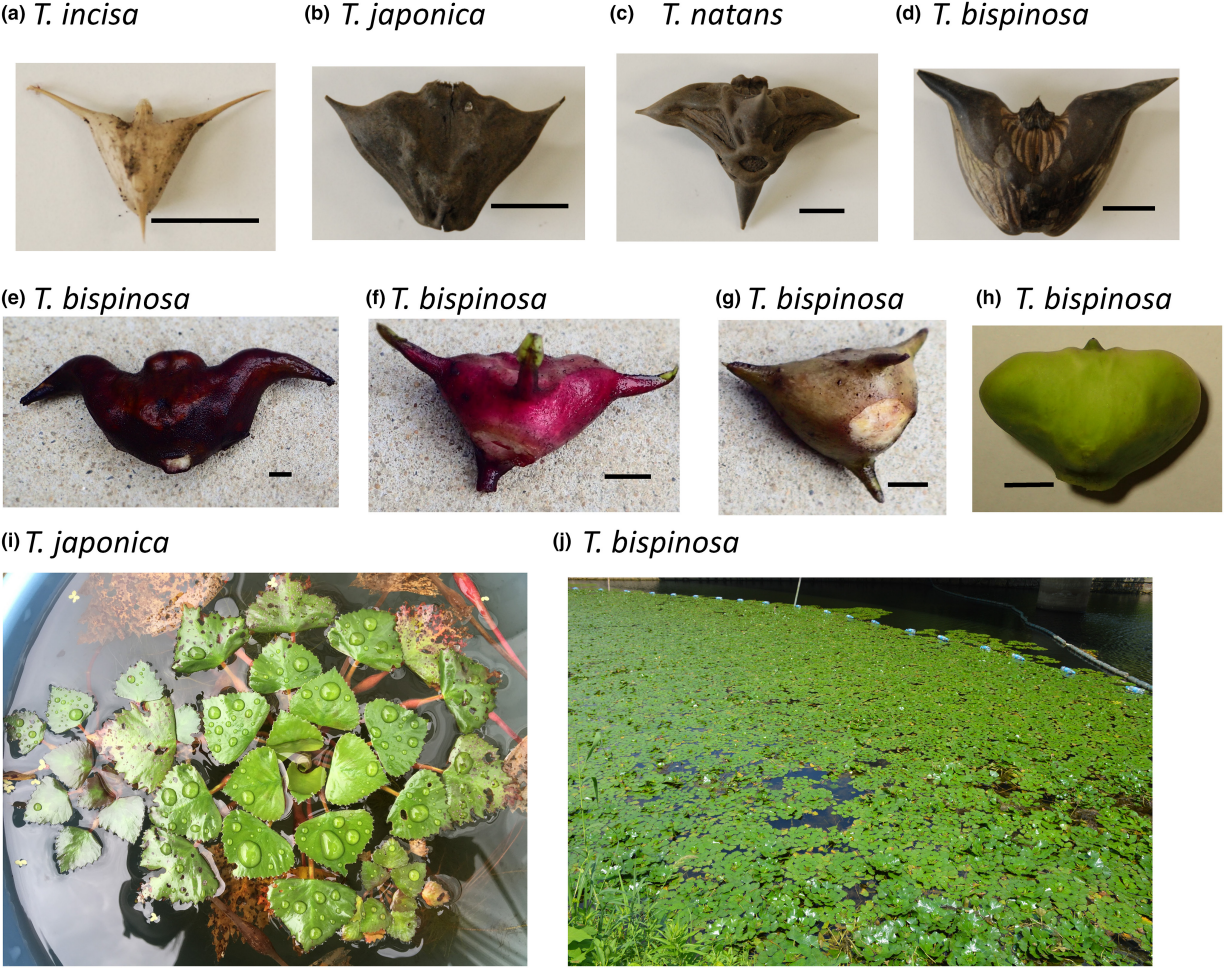
Appearances of seeds among *T. incisa* (a), *T. japonica* (b), *T. natans* (c), and *T. bispinosa* (d). (e–h) photos of domesticated species *T. bispinosa* collected in farmer's field in Yoyao, Liangzhu and commercial markets in Nanjing (i) A clump of *T. japonica*. (j) Vegetation of *T. bispinosa* cultivated in Lake Taifu at Jiangsu, China. Scale bars = 1 cm in the first eight panels.

**FIGURE 2:**
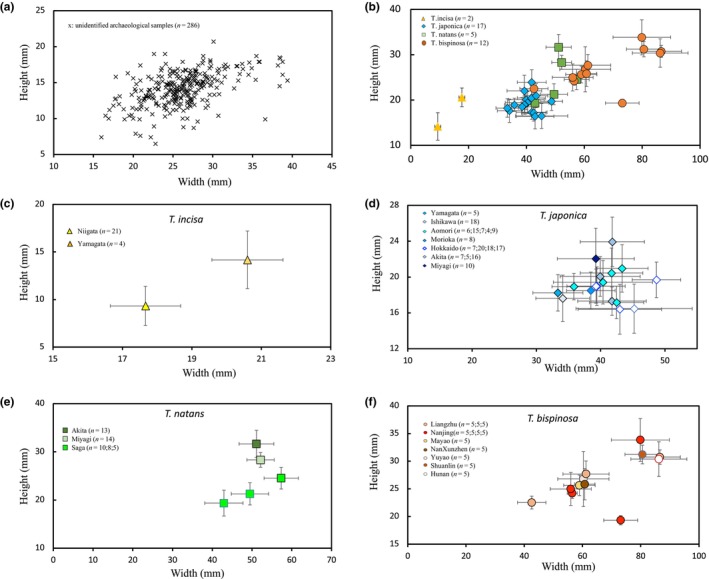
Morphological variations in seed size among *Trapa* species. (a) Seed size variation among unidentified archeological *Trapa* seeds excavated at the Tianluoshan site. (b) Seed size variations among wild and domesticated *Trapa* species. The values are taken from the mean value for each species. Triangles indicate *T. incisa*, diamonds indicate *T. japonica*, squares indicate *T. natans*, and circles indicates *T. bispinosa*. Number of dots indicate number of populations. (c) Variations in seed size among *T. incisa* collected in Niigata and Yamagata, Japan. (d) Variations in seed size among *T. japonica* collected in Yamagata, Ishikawa, Aomori, Morioka, Hokkaido, Akita, and Miyagi, Japan. (e) Variations in seed size among *T. natans* collected in Akita, Miyagi and Saga, Japan. (f) Variations in seed size among *T. bispinosa* collected in Yuyao, Liangzhu (with two green spines and four green spines types), Nanjing (with white‐colored landraces with four spines, white‐colored landraces with two spines, black‐colored landraces with two spines, black colored with two downward spines types), Hunan, Shuanglin, Mayao, and NaXunzhen, China. The bars indicate SD values.

Three wild species including *T. incisa*, *T. japonica*, *T. natans*, and domesticated species, *T. bispinosa* collected both in China and Japan showed diverse in seed appearance, especially the *T. bispinosa* collected from cultivated farmers' fields and local markets (Figure 1). Measuring of seed capsules indicating *T. incisa* has the smallest seed size (Figure 2b). The largest seed size was shown in *T. bispinosa*, although with some variation. *T. natans* had medium size, partial overlap with *T. bispinosa* and *T. japonica*. Archeological samples were also used for seed measurement (Figure 2a), showing seed's height varied from 5 to 25 mm, and seed's width varied from 10 to 45 mm, close to the size of *T. incisa* and *T. japonica*. Details of variation in seed size in each species are shown in Figure 2.

**FIGURE 3:**
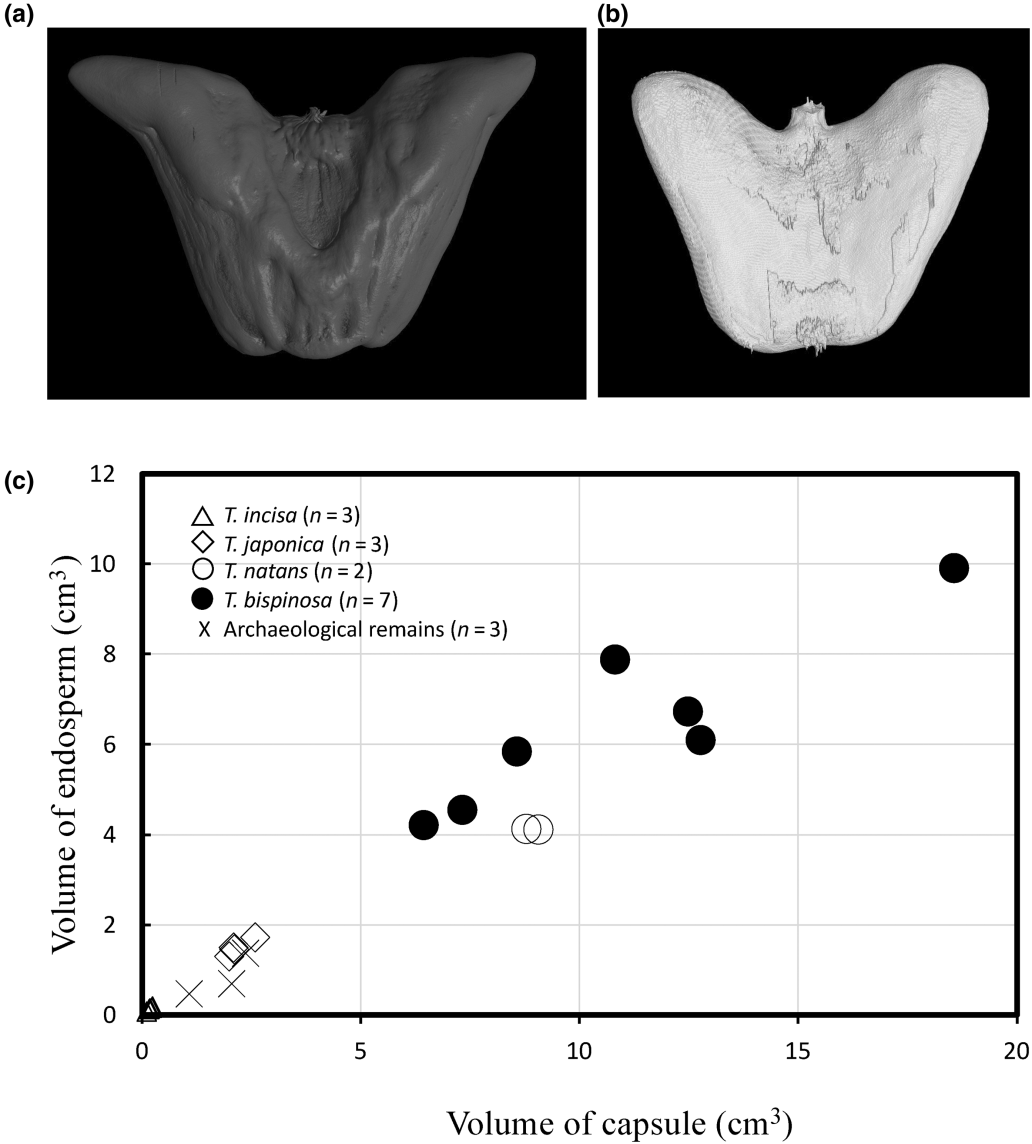
Three‐dimensional data of seed capsule and endosperm volumes to evaluate size variations among wild and domesticated species. (a) Three‐dimensional image of a seed capsule of *T. bispinosa*. (b) Three‐dimensional image of the inner endosperm. (c) Variation in volumes among modern samples from four species and archeological samples. Triangles indicate *T. incisa*, diamonds *T. japonica*, open circles *T. natans*, solid circles *T. bispinosa*, and X marks were archeological samples.

Seeds of two species, *T. japonica* and *T. bispinosa*, were compared using X‐ray computed tomography (CT) scans (Figure 3). Ratios of endosperm per total volumes ranged from 53% to 68.9% in which higher ratio went to *T. japonica*. The total volumes of *T. bispinosa* and two *T. japonica* species ranged from 4.2 to 4.5, and ratios of endosperm per total volume ranged from 3.3 to 3.7. It demonstrated the domesticated type, *T. bispinosa*, had larger seed volumes than wild species. It also suggested that the domesticated species had larger volumes than archeological samples, which were collected and used as edible resources at approximately 7000–6300 cal BP at the Tianluoshan site.

### 3.2 Ploidy level

Nuclear DNA content was assessed using flow cytometry with the different species (Table 1). Rice nuclei were added as an internal control because the genome size has been determined as 373,245,519 nucleotides (nt) in the haploid genome (1C). The relative migration distance of each water chestnut compared to that of rice was used to estimate its genome size. Then, *T. natans* and *T. incisa*, which are diploid (2n = 48), have 438,297,965 nt and 406,826,068 nt, respectively. In contrast, the nuclear content of *T. japonica* (2n = 96) was 883,068,853 nt, which is nearly double the other two species, corresponded to the difference in chromosome number. *T. bispinosa* cultivated in Japan showed 481,616,741 nt. Two *T. bispinosa* accessions collected in China ranged from 448,974,828 nt to 481,537,585 nt. Although the number of chromosomes has not been verified, its genome size inferred from other species would be diploid.

**TABLE 1:** Estimated haplotype genome size by flow cytometry, with *Oryza sativa* cv. Nipponbare as an internal control.

Plant species	Collection site	Peak	Internal peak^a^	Genome size (bp)
*Oryza sativa* cv Nipponbare	–	–	–	37,32,45,519
Collected in Japan				
*Trapa bispinosa*	Oki village, Fukuoka	161.50	125.16	48,16,16,741
*Trapa incisa*	Fukui	115.55	98.40	43,82,97,965
*Trapa japonica*	Hirosaki	289.21	122.24	88,30,68,853
*Trapa natans*	Kanzaki city, Saga	133.87	122.82	40,68,26,068
Collected in China				
*Trapa bispinosa*	Liangzhu, 4 spines, green	195.35	162.40	44,89,74,828
*Trapa bispinosa*	Liangzhu, 4 spines, red	254.66	197.39	48,15,37,585
*Trapa bispinosa*	Liangzhu, 2 spines, red	258.66	204.49	47,21,19,350
*Trapa japonica*	Ninpo	409.47	187.44	81,53,69,412

^a^ Internal peaks were obtained with *Oryza sativa* cv. Nipponbare.

### 3.3 Reconstructed chloroplast genome

**FIGURE 4:**
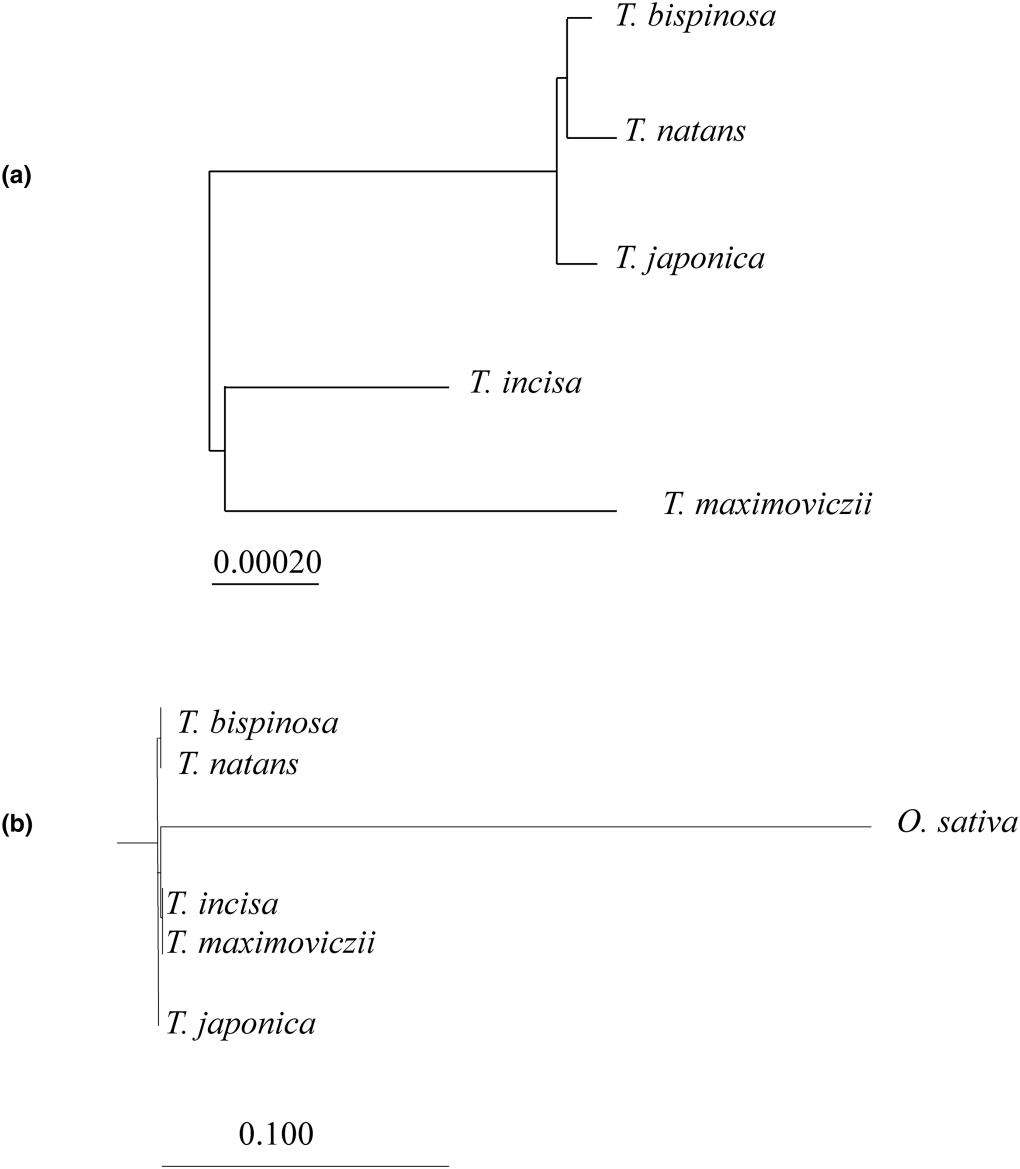
Phylogenetic tree was drawn using the neighbor‐joining method. (a) Phylogenetic tree with 198 informative nucleotide variations in whole chloroplast genomes among five species, *T. incisa*, *T. japonica*, *T. natans*, and *T. bispinosa*. The bar shows genetic distance. (b) Phylogenetic tree with parts of short single copy (SSC) sequence among the four *Trapa* species and rice genome sequence *O. sativa* as control. The corresponding sequence of rice was obtained from a complete chloroplast genome XP15901, where relatively high homology sites from 105,059 to 113,727 nt. Rice and *T. maximoviczii* shared 88.2% homology without gaps in which 5945 nt was not attempted and 2652 nt was matched with each other. Bar showed tree distance by the method of ML calculated by CLC workbench genomics software Ver.8.

Contigs obtained by de novo assembly were screened for average coverage and consensus length. *T. japonica* generated 88,699 nt with 2675 average coverage, 24,386 nt with 5350 average coverage, and 18,421 nt with 3271 average coverage. These contig sequences showed higher similarity with chloroplast sequences of *Oryza sativa* cv. Nipponbare. *T. bispinosa* generated 88,684 nt with 3661 average coverage, 24,452 nt with 7313 average coverage, and 18,414 nt with 4296 average coverage, which corresponded to the large single copy (LSC), inverted repeat (INV), and small single copy (SSC) regions with some flanking sequences. De novo assemblies of *T. natans* and *T. incisa* tended to generate parts of chloroplast genomes with higher coverage. In general, chloroplast genomes carry two inverted repeats, and these contigs were found to form one single circular molecule carrying two inverted repeats. Resequencing analysis was also attempted for the whole chloroplast genome of *T. maximowiczii* (Xue et al., 2017). The presumed chloroplast genomes reconstructed using contigs were reconfirmed. Whole chloroplast genomes were 155,534 nt in *T. bispinosa*, 155,575 nt in *T. japonica*, 155,519 nt in *T. incisa*, and 155,531 nt in *T. natans*. The data were applied to construct a phylogenetic tree. The result showed *T. bispinosa* shared the highest similarity with *T. natans*, next to *T. japonica*, while *T. maximowiczii* and *T. incisa* were distant from *T. bispinosa* (Figure 4).

### 3.4 Maternal lineages

The presumed long single circular regions were aligned to detect discrepancies between the contigs. Potential INDEL polymorphisms between *T. bispinosa* and *T. japonica* were detected (Table 2). Ten cpINDEL markers were developed based on single nucleotide repeats. One complex structure was detected as single T nucleotide repeats and tandem duplications. Four INDELs were composed of tandem repeats inside the sequences. One variation included an SSR. Three INDELs did not show any obvious structures. All detected INDELs were confirmed using polymerase chain reaction and subsequent electrophoresis among different accessions. Five of contig10‐31cp INDELs failed to amplify probably caused by the primer designs. The remaining five INDELs were monomorphic suggesting rare alleles were detected and applied to next‐generation sequencing (NGS). Another nine INDELs were polymorphic among the examined materials, as shown in Table 3. Combinations of the INDEL genotypes comprised 12 lineages as plastid types. Two lineages were termed as *incisa A* and *incisa B*. They were detected only in *T. incisa*. Type W was observed in *T. japonica* samples from both China and Japan, and one *T. natans* from China. Eight lineages were detected among *T. natans* and *T. bispinosa*.

**TABLE 2:** Chloroplast INDEL markers developped in this study, from alignment results between two reconstructed chloroplast genomes of *Trapa bispinosa* and *Trapa japonica*.

Locus	Primer sequence		Core sequences including variation	Motif	Primer position in KY705084		
	Forward	Reverse			Start	End	Length
cpINDEL1	GTTCGATACACTGTTGTCAATATG	CTAACAAAGAAATAAACCCGAG	Plastid type	Single nucleotide repeat	4727	4891	165
cpINDEL2	AATTTCGTCGGCTCTAATGG	AGTTACATTTACGTACCATC	TATTATTATTA‐‐‐‐‐‐‐TTTTTTTTTTAAATAA TATTATTATTATTTTTATTTTTTTTTTTAAATAA	Single nucleotide repeat	12,533	12,694	162
cpINDEL3	TAGAGTTCTTTTTGTATCAC	TATTCCGTAATTGATTGACA	TTTCTATTTATTTCTATTTAAA//ATTTTTTT‐TTTTCA TTTCTATTTA‐‐‐‐‐‐‐‐‐‐AA//ATTTTTTTTTTTTCA	Tandem/Single nucleotide repeat	13,987	14,168	182
cpINDEL4	GGATATGTGGTATAAAAATGGAG	CTTCACGTCCAGGATTACGTCC	CTTTTTTTTTTTTTAAAAAAAAAAAA‐‐‐‐TGATC C‐‐‐‐‐‐‐‐TTTTTAAAAAAAAAAAAAAAATGATC	Single nucleotide repeat	8331	8514	184
cpINDEL5	CACGTGTATGAATTCGTTAGG	ATTTTCTTTTGTTCAATTGAACAAAAG	TATTTTTTTTTTTTTTTTTTTATTATTATTTATTC TATTTTTTTTTTTTTTTTTTT‐‐TATTATTTATTC	Single nucleotide repeat	15,341	15,495	155
cpINDEL6	ATTGTAACAGAGGTGCAAGTG	GTATAAAGTGGAAGGGAAAGAGAG	TGGGTGGGGTGGGTACTTT//G‐AAAAAAAAAAAC TGGG‐‐‐‐‐TGGGTACTTT//GAAAAAAAAAAAAC	Single nucleotide repeat	32,643	32,810	168
cpINDEL7	GTATACTCATACTAGCTATAAC	TTCAAGAGACGGCTTATCTATC	AAATAAAGATTTCAACCTTTTTTTTTTTATTCTAA AATAAAGATTTCAACC‐‐TTTTTTTTTTATTCTAA	Single nucleotide repeat	34,025	34,159	135
cpINDEL8	GAATAGGTGGGTCAATTCCTTCC	CAAAATGGGATATACCTATGAG	CCCATTTTTTTT‐‐‐ATTTACCCTATCTAATTGAA TCCCATTTTTTTTTTATTTACCCTATCTAATTGAA	Single nucleotide repeat	45,032	45,178	147
cpINDEL9	GATCGAATCAAGATGTGTCAC	AATTGAAGTTCTATTTCTAAGTTC	GTTTAGAAATACTATAAATACTATATTAGATATTA GTTTAGAAATACTATA‐‐‐‐‐‐‐‐‐‐‐‐‐‐‐‐TTA	Tandem	71,742	71,910	169
cpINDEL10	TCCCTCTTCCATTTATCTGCATAC	GGACCTACCCATACTATGAAC	AAAGCAGATTGGTTTTT‐‐‐‐‐‐‐‐‐TTTATTTTA AAAGCAGATTGGTTTTTCTTTTGTTTTTTATTTTA	No motif	86,751	86,955	205
cpINDEL11	GAATCGTAATGCTATGTACATTATC	TGAGATCCGATAGCTAGTATGG	TATACAGTATATTAT‐‐‐‐GGTATTGTTACATTAC TATACAGTATATTATGTATGGTATTGTTACATTAC	Tandem	30,898	31,076	179
cpINDEL12	GGGAAAAACGGGGATATTAGTG	TTCAAGAGACGGCTTATCTATC	AAATAAAGATTTCAACCTTTTTTTTTTTATTCTAAA AATAAAGATTTCAACC‐‐TTTTTTTTTTATTCTAAA	Single nucleotide repeat	33,967	34,159	193
cpINDEL13	TAGCATTGGAACTGCTATGTAGG	GGAACCACGGGAGGAATAGTG	TGGTTCAAGGCGT‐‐‐‐AGCATTGGAACTGCTTGTA TGGTTCAAGGCGTACCAAGCATTGGAACTGCTTGTA	No motif	47,208	47,398	191
cpINDEL14	CATAGATGAACTCCTATGAATG	CTTGAAATGAGACATGTACC	AGTCGATTAAATT‐‐‐‐‐‐‐‐TTGTCAAGCCATCCAT AGTCGATTAAATTGGAATTAATTGTCAAGCCATCCAT	Tandem	53,227	53,381	155
cpINDEL15	TAGCTATAGAGTATCATATC	AAAATCCATTCTTGTCTTATC	CAACAAGATAAA‐AAAAAAAAAAGAAAATCCTGCCTT CAACAAGATAAAAAAAAAAAAAAGAAAATCCTGCCTT	Single nucleotide repeat	62,375	62,542	168
cpINDEL16	CTTACACTTACTTCGACTTAG	TTTCGTTGCAATCACAACCC	TAAAGTAAAGTTAAGTAAAGTAATAATAATATAGTAATATTA T‐‐‐‐TAAAGTTAAGTAAAGTAATAATAATATAGTA‐‐‐‐‐A	SSR	71,159	71,408	250
cpINDEL17	ATTCACAAGAACCGCGAATTC	CATTATAACAAGTCACACACTC	AACCGCGAATTCTT‐‐‐‐‐ATTAATACTTTTATACTTA AACCGCGAATTCTTACTTTATTAATACTTTTATACTTA	No motif	78,991	79,205	215
cpINDEL18	ACTATTTATCTTAATATGGTTGT	TTAAGTTATTACTCACAACAAAG	TAATATAAATATAGAAAAGTAAAAA‐‐‐‐‐CCTATTG TAATATAAATATAGAAAAGTAAAAATTTTTCCTATTG	Single nucleotide repeat	123,167	123,346	180
cpINDEL19	ATTCTTTCTTGTTCGTATTTTG	ACTTTGCTAAAAAATGACATAG	TTTGAAAATCCAATCCAAATAAAGTATTTTCTCTTGA TTTGA‐‐‐‐‐‐AATCCAAATAAAGTATTTTCTCTTGA	Tandem	130,558	130,730	173

**TABLE 3:** Variation in cpINDEL among wild and domesticated types of water chestnuts in China and Japan.

Plastid type	Region	cpINDEL									Number of accessions			
		2	3	7	9	10	12	13	17	19	*T. incisa*	*T. japonica*	*T. natans*	*T. bispinosa*
incisa A	Japan	5	1	1	1	2	2	2	1	1	2			
incisa B	Japan	2	3	3	1	2	2	1	2	2	2			
W	Japan	1	3	2	2	1	3	2	1	1		5		
W	China	1	3	2	2	1	3	2	1	1		11		
natans‐bispinosa 1	China	1	2	2	2	1	2	2	1	1			5	
natans‐bispinosa 2	China	2	1	1	1	2	1	2	1	1			1	6
natans‐bispinosa 3	China	2	1	2	1	2	2	2	1	1				2
natans‐bispinosa 4	Japan	3	1	1	1	2	2	2	1	1			3	4
natans‐bispinosa 5	China	3	1	1	1	2	2	2	1	1			1	5
natans‐bispinosa 6	China	3	2	2	1	2	2	2	1	1				3
natans‐bispinosa 7	Japan	4	2	1	1	2	1	2	1	1				4
natans‐bispinosa 8	China	4	2	1	1	2	1	2	1	1				25

*Note*: Combinations of the cpINDEL genotypes comprised 12 lineages as plastid types.

## 4 DISCUSSION

Water chestnut *T. bispinosa* is a well‐known seasonal food in the autumn season in China. A recent publication reported that large volumes of rice and water chestnuts were excavated along with fish bones and other animals from archeological sites from the period approximately 7000–6300 cal BP (Guo et al., 2017). People living in the wetland developed rice cultivation in paddy fields and yielded sufficient crops. In addition, there were abundant remains of water chestnuts on the site. However, the seed size was much smaller than the present domesticated species. The wild species is still colonized as one of the weeds suggested by de Wet and Harlan (1975). Humans disturbed wild colonizers through man‐made habitats such as upland crops for food resources and rice cultivated in upland fields or paddy fields (Fuller, 2011; Nakamura, 2010; Guo et al., 2017). If the general food supply is sufficient, wild species used as edible resources may be easily abandoned. In fact, during World War II and the post‐war period, wild water chestnuts were collected to complement daily food supplies in Japan. At present, some trials to cultivate the tetraploid species are being tried, but the most limited factor is the seed volume. The yield of a domesticated type, *T. bisponosa* was about 55–100 kg/10a, but one of *T. japonica* was 25–40 kg/10a (Hyakusima & Nakamura, 1974; Nakamura, 2004). As suggested by CT scans, the size of domesticated species is larger than that of tetraploid species. In general, there are two ways to enlarge the volumes of seeds: (1) increase ploidy level or (2) induce mutations in genes regulating seed size. To ensure how artificial selection had affected to *T. bispinosa*, flow cytometry was applied to ensure the ploidy level of the domesticated species. Our study concluded that *T. japonica* is tetraploid, while *T. bispinosa* is diploid which is similar to other diploid species in the *Trapa* genus. It suggested that the larger fruit size of *T. bispinosa* may result from artificial selection but not from polyploidization.

In the previous report (Guo et al., 2017), only small capsules of water chestnuts were found. The size of water chestnuts, however, was not like current domesticated species in size. Thus, human selection against the size change has been done during several thousand years since then. Such long term is required to expect as domestication process (Purugganan & Fuller, 2009). Phylogenetic data suggested that *T. natans* is the closest wild species to the domesticated species. *T. natans* has a similar seed size to domesticated species but also has a relatively smaller seed endosperm and hard outer capsule. It suggested us that seed size and thickness of the capsule would be targeted for artificial selection over 6000 years. On the other hand, although *T. japonica* is tetraploid, the seed size is not larger than other diploid species. It might be originated from its parental species *T. incisa* (Takako & Kadono, 2005), suggesting size limitation from parent works for the medium seed size of the tetraploid species. Variation of capsules in the domesticated species are observed in the collected sites. However, we have not seen such variation in Taiwan, Thailand, and Vietnam. It is not only these observations but also restriction of archeological reports involving to *Trapa* species. It is suggesting that the middle of East Asia is one of center of diversity and probable site where artificial selection happened.

**FIGURE 5:**
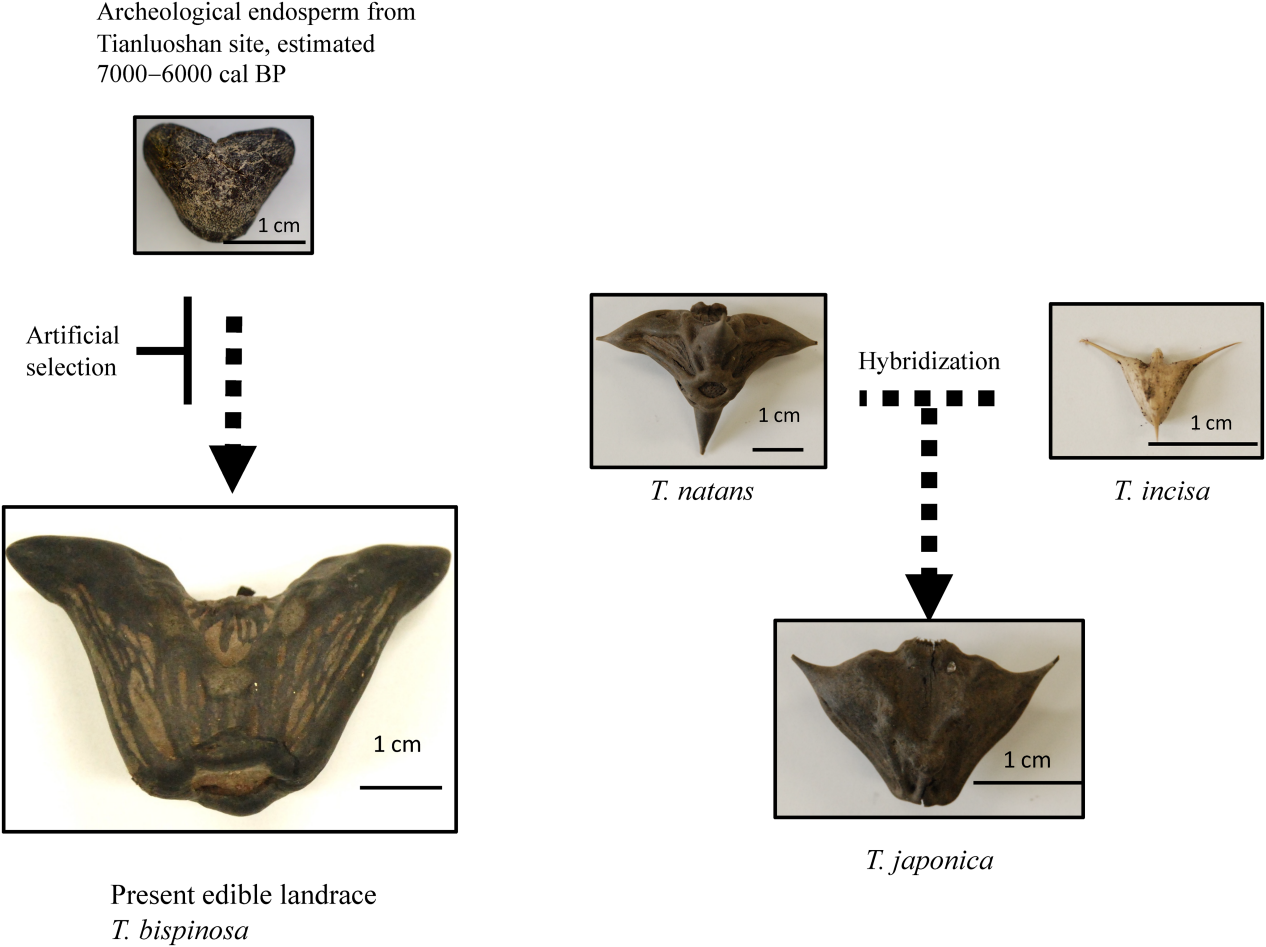
Model of the evolutionary process of the *Trapa* genus. Chloroplast genome sequences and cpINDELs genotyping proposed *T. natans* would be the maternal donor of *T. japonica*. The medium seed size of *T. japonica* could be inherited from *T. incisa* limiting the size despite the tetraploid type. Domesticated water chestnuts *T. bispinosa* has larger seed sizes and thinner capsules than the wild species. Archeological specimens collected as food resources and remained as inner starch endosperm were exposed for artificial selection over more than 6000 years and has probably been based on large seed size and thin capsule.

Chloroplast genome sequences were completed using NGS reads, ranging from 155,519 nt in *T. incisa* to 155,575 nt in *T. bispinosa*. A previously reported genome sequence of *Trapa* genus is available as 155,577 nt for *T. maximowiczii* Korsh (Xue et al., 2017). The sequence of *T. bispinosa* suggested that the species is close to *T. natans* through phylogenetic analysis based on SNPs among genomes (Table S4). Further evidence comes from molecular markers applied in this paper identifying maternal lineages indicating both wild species in China and Japan shared an identical plastid type to *T. japonica*. These phylogenetic relations were suggested with whole chloroplast genome sequences by Fan et al. (2022). Karyotypes (Oginuma et al., 1996) inferred the species might be generated by interspecies hybridization resulting from duplication of their genomes like wheat genomes. However, among accessions tested, *T. japonica* was close to *T. natans*. A model of polyploidization is proposed that the maternal donor of *T. japonica* would be *T. natans*. Then, tiny seed such as *T. incisa* would be paternal donors to result in medium seed size of *T. japonica*. We may not have enough samples to trace and cover all maternal and paternal lineages in Trapa species. Nevertheless, the morphological and genotype results could be enough to make us conclude that domesticated types shared high similarity with wild species, *T. natans* not only has the complete chloroplast sequence but also cpINDELs. The seed size and volumes of endosperm were similar between the two species, although the domesticated type has larger seed size than the wild species. Archeological specimens have not shown large seeds as seen in the domesticated type. Artificial selection over more than 6000 years has probably made the seed size larger and the capsule thinner. Based on these findings, we developed a model of how domestication occurred in the *Trapa* genus and how ploidy changes happened (Figure 5). To make the phylogenetic relationships among the wild species clearer, wider genetic resources covering all areas in East Asian countries are needed because there were no lineages sharing the same maternal plastid type with *T. japonica*.

Domesticated water chestnuts are dispersed over a relatively wide area. Japanese water chestnuts were introduced from Nanjing, China, to Japan in the 1920s. The descendants shared an identical plastid type with one of the landraces which are still cultivated in Nanjing. The molecular markers developed in this study are available to trace lineages in water chestnuts, which detected multiple plastid types among *T. bispinosa*. The information enabled us to determine the dispersal pattern and the origin of domesticated types of water chestnuts in China. Nuclear marker tools will be necessary to determine the pattern in detail.

Parts of chloroplast genes have been applied for taxonomic evaluation of Trapaceae but not within species in detail because of a lack of variation within genus or species (Conti et al., 1997; Graham et al., 2005). In this study, cpINDELs developed using NGS data are made available to resolve differences among species. Other molecular markers have been developed to identify variation within Trapaceae (Jiang & Ding, 2004; Kim et al., 2010; Takano & Kadono, 2005). In some cases, species identification was presumed, but there were not enough markers for conclusive evaluations. Reliable sequence‐tagged molecular tools are required in order to understand clear phylogenetic analysis. Here, we developed cpINDELs based on complete chloroplast sequences obtained using NGS technology. This approach is available for other plant species with de novo methods. Other contigs also supplied possible SSRs. These new tools that can be shared among researchers for application in complex species models such as Trapaceae.

## 5 CONCLUSIONS

Whole chloroplast genome sequences suggested that *T. natans* could be a maternal donor of *T. japonica*. Domesticated water chestnuts *T. bispinosa* shared high similarity with wild species, *T. natans* in complete chloroplast sequences and cpINDEL genotypes. Their seed size and volumes of endosperm were similar between the two species, although the domesticated type had a larger seed size and thinner capsule than the wild species. Archeological specimens show small seeds indicating artificial selection over more than 6000 years has probably made the seed size larger and thinner capsule.

## AUTHOR CONTRIBUTIONS

**Dinh Thi Lam:** Data curation (equal); investigation (equal); methodology (equal); visualization (equal); writing – original draft (equal); writing – review and editing (equal). **Taro Kataoka:** Methodology (equal). **Hiroki Yamagishi:** Investigation (equal). **Guoping Sun:** Resources (equal). **Tetsuro Udatsu:** Data curation (equal). **Katsunori Tanaka:** Funding acquisition (equal); investigation (equal). **Ryuji Ishikawa:** Conceptualization (equal); data curation (equal); formal analysis (equal); genome analysis, flowcytometer analysis, funding acquisition (equal); investigation (equal); methodology (equal); project administration (equal); resources (equal); software (equal); supervision (equal); validation (equal); visualization (equal); writing – original draft (equal); writing – review and editing (equal).

## FUNDING INFORMATION

This work was funded by a Grant‐in‐Aid project No. 15H0596 and partly by a Grant‐in‐Aid for Scientific Research on Innovative Areas (15H05968).

## CONFLICT OF INTEREST STATEMENT

None declared.

## Supporting information


Data S1

## Data Availability

All data generated or analyzed during this study are included in this published article and its supplementary information files.
